# Trends in the burden of road traffic injuries among children and adolescents aged 0–19 years in low- and middle-income countries, 1990–2023

**DOI:** 10.7189/jogh.16.04094

**Published:** 2026-03-13

**Authors:** Zhe Song, Bing Zhang, Sihan Pang, Mingliang Qiu, Jiang Huang, Junke Hao, Xiao Yang, Yong Li

**Affiliations:** 1 Department of Intensive Care Medicine, The Affiliated Hospital of Yangzhou University, Yangzhou University, Yangzhou, China; 2 Department of Emergency Medicine, The Affiliated Hospital of Yangzhou University, Yangzhou University, Yangzhou, China; 3 Hong Kong University of Science and Technology (Guangzhou), Thrust of Urban Governance and Design, Society Hub, Guangzhou, China

## Abstract

**Background:**

Road traffic injuries remain a leading cause of death and disability among children and adolescents worldwide, particularly in low- and middle-income countries (LMICs), where rapid motorisation and limited trauma care capacity increase vulnerability. In this study, we aimed to characterise long-term patterns and potential future trajectories of the burden of road traffic injuries among children and adolescents aged 0–19 years in LMICs, using estimates from the Global Burden of Disease (GBD) 2023 study.

**Methods:**

From the GBD 2023 database, we extracted incidence, prevalence, mortality, and disability-adjusted life years (DALYs) for road traffic injuries across 129 LMICs and stratified them by age, sex, and gross national income. We assessed temporal patterns using estimated annual percentage change and joinpoint regression. Further, we used decomposition analysis to illustrate the relative contributions of population growth, age structure, and epidemiological change to the disease burden. We used autoregressive integrated moving average (ARIMA) models for exploratory and scenario-based projections of future trends.

**Results:**

Between 1990 and 2023, the overall burden of road traffic injuries among children and adolescents in LMICs declined across DALYs, mortality, incidence, and prevalence. Declines were most pronounced in upper-middle-income and more modest in low-income countries. Motor vehicle-related injuries accounted for the largest share of DALYs across income groups. Males and older adolescents showed higher estimated rates and slower declines. Decomposition analysis indicated that population growth was the primary driver of the increasing burden in low-income countries, whereas epidemiological improvements were primarily observed in upper-middle-income countries. Exploratory extrapolations of ARIMA suggested that DALYs and mortality might continue to decline, while incidence and prevalence might stabilise or increase modestly under unchanged historical trends.

**Conclusions:**

Based on estimates from GBD 2023, the burden of road traffic injuries among children and adolescents in LMICs has declined over the past three decades, despite substantial differences across different income, age, and sex groups. These findings should be interpreted as estimated patterns rather than directly observed epidemiological changes. Strengthening road safety, trauma care, and prevention strategies, particularly in low-income settings, is essential to reduce inequality and mitigate the burden of road traffic injuries in children and adolescents.

Road traffic injuries are a major global public health concern, imposing substantial health, social, and economic burdens [1,2]. Children and adolescents (aged <20 years) are particularly vulnerable to road traffic injuries-associated severe physical trauma and long-term psychological and social consequences [3]. The burden is disproportionately concentrated in low-income countries due to inadequate infrastructure, limited healthcare capacity, and weaker road safety regulations [1,4].

Most previous studies have focused on global-level or disease-specific estimates based on the Global Burden of Disease (GBD) database, while systematic analyses for low- and middle-income countries (LMICs), especially low-income countries, remain scarce [5]. Few studies have examined the heterogeneity of the burden of road traffic injuries based on demographic characteristics or epidemiological transitions, and national or age-specific patterns are rarely explored [6]. Given the complex traffic environments and limited medical resources in LMICs, this gap underscores a critical research need [7].

In this study, we aimed to analyse trends in the burden of road traffic injuries among children and adolescents (aged 0–19 years) in LMICs based on the GBD 2023 database. We conducted gross national income (GNI)-based correlation analyses to assess the impact of national income, and implemented a decomposition analysis to quantify the contributions of demographic and epidemiological changes. We adopted an autoregressive integrated moving average (ARIMA) model to forecast trends through 2050, providing evidence for targeted policy development.

Compared with previous GBD-based studies, this study provides original insights in two main respects. First, in addition to reporting temporal changes, we integrated joinpoint regression and decomposition analysis to quantify the relative contributions of population growth, age structure, and epidemiological change to the observed disease burden. Second, we focused specifically on LMICs and the pediatric population (aged 0–19 years) to evaluate inequalities in the burden of road traffic injuries and their potential policy implications. These methodological improvements could enhance the understanding of how demographic transitions and differences in health systems shape the burden of road traffic injuries in LMICs.

As a reanalysis of secondary data, our study adhered to the Journal of Global Health Guidelines for Reporting Analyses of Big Data Repositories Open to Public (GRABDROP) (Table S1 in the **Online Supplementary Document**) [8].

## METHODS

### Data source and study design

The GBD database is an international collaborative project that provides standardised, comparable estimates of health loss from major diseases and injuries across countries and time [9]. It integrates data from epidemiological studies, health surveys, vital registration systems, and administrative records using uniform statistical modelling to ensure consistency. The GBD framework estimates incidence, prevalence, mortality, and disability-adjusted life years (DALYs), enabling systematic assessment of disease burden globally.

The GBD database provides modelled estimates derived from heterogeneous data sources rather than directly observed data. Therefore, the reported trends should be interpreted as model-based estimates of health burden with associated uncertainty, particularly in low-income countries where primary data are limited.

In this study, we used estimated annual percentage change (EAPC) and joinpoint regression as the primary methods to evaluate long-term temporal trends. We applied percent change (PC) and correlation analyses as supplementary descriptive tools. Further, we conducted decomposition analysis to partition the relative contributions of demographic and epidemiological changes. ARIMA was used for scenario-based rather than deterministic projections. Using the Global Health Data Exchange (GHDx) Results Tool (Institute for Health Metrics and Evaluation, Seattle, Washington, USA) we extracted estimates and rates for incidence, prevalence, mortality, and DALYs with 95% uncertainty intervals (UIs), stratified by age (<5, 5–9, 10–14, 15–19, and <20 years), sex (female, male, both), region (three GNI strata, 129 countries), cause (road injuries), and year (1990–2023) [9,10].

### Definitions and classification

We defined road traffic injuries according to the World Health Organization (WHO) classification as traffic-related physical trauma involving motor vehicles, bicycles, or pedestrians, identified by the International Classification of Diseases ninth revision (ICD-9) codes E810–829 and ICD-10 codes V01–89. Further, based on the WHO criteria, we defined children and adolescents as individuals aged 0–19 years [11]. National income was measured using GNI *per capita* [12] and categorised as low (GNI-L), lower-middle (GNI-LM), or upper-middle income (GNI-UM) following World Bank classification [13,14]. We estimated DALYs as the sum of years of life lost and years lived with disability [13,15].

We used publicly available data, which did not require ethical approval. We followed the GATHER reporting guidelines [16] and used comprehensive analytical methods (trend metrics, decomposition, and scenario-based projections) to examine the contributions of demographic and epidemiological changes to disease burden across income levels.

### Statistical analysis

We utilised multiple analytical approaches to evaluate temporal trends and forecast future burden. We calculated EAPC and PC to quantify trends over time [17]. Further, we performed joinpoint regression using permutation-based testing, which inherently adjusted for multiple comparisons. Moreover, we used joinpoint regression to identify significant trend inflexion points and estimate annual percentage changes (APCs) within each segment [18]. We performed decomposition analysis to assess the relative contributions of population growth, ageing, and epidemiological changes to variations in burden [9].

### Forecast modelling

We projected future trends to 2050 using ARIMA models, assuming historical patterns would persist. These projections were scenario-based and did not account for structural changes such as policy, technological, or demographic changes. Future trends in mortality and DALYs were projected using an ARIMA model that integrates autoregressive, differencing, and moving-average components. Model selection was guided by the Akaike information criterion and the Bayesian information criterion values, and the Ljung-Box Q test confirmed the residuals were white noise, indicating good model fit [19]. These projections should be interpreted as exploratory findings, assuming that historical patterns will persist.

For all analyses, except age-period-cohort modelling in joinpoint, we used *R*, version 4.5.0 (R Core Team, Vienna, Austria). To control for multiplicity arising from multiple subgroup and trend analyses, we applied the Benjamini-Hochberg method to control the false discovery rate (FDR). An FDR-adjusted q-value <0.05 was considered statistically significant. For analyses unsuitable for FDR adjustment (*e.g.* joinpoint permutation tests), two-sided *P*-values <0.05 indicated statistical significance, and the consistency of results across methods and UIs was considered.

## RESULTS

### Trend analysis

Between 1990 and 2023, the global burden of road traffic injuries declined substantially. The DALY rate declined from 1211.28 per 100 000 population (95% UI = 780.02, 1707.71) to 770.46 per 100 000 population (95% UI = 510.45, 1082.2), with an estimated annual percentage change (EAPC) of −1.51 (95% UI = −1.56, −1.46). The mortality rate fell from 14.84 per 100 000 population (95% UI = 9.42, 21.07) to 9.65 per 100 000 (95% UI = 6.35, 13.58), with an EAPC of −1.45 (95% UI = −1.50, −1.40). The incidence rate diminished from 835.19 per 100 000 (95% UI = 707.53, 971.03) to 386.99 per 100 000 (95% UI = 325.92, 453.9), with an EAPC of −2.47 (95% UI = −2.56, −2.39). The prevalence rate decreased from 563.69 per 100 000 (95% UI = 509.14, 624.68) to 228.74 per 100 000 (95% UI = 208.97, 251.28), with an EAPC of −3.03 (95% UI = −3.15, −2.90). Overall, all four indicators suggested a continuous downward trend, with the most considerable reductions observed in prevalence and incidence rates.

At the GNI level, GNI-UM countries exhibited the steepest declines, with EAPCs for DALY rate, mortality rate, and prevalence rate ranging from –2.8% to –3.5%. GNI-LM countries showed a stable decline, with the DALY rate dropping from 905.82 per 100 000 (95% UI = 578.84, 1282.45) to 616.59 per 100 000 (95% UI = 396.23, 891.09), with an EAPC of −1.27 (95% UI = −1.35, −1.18). In contrast, GNI-L countries experienced only modest reductions, with a DALY rate EAPC of −0.89 (95% UI = −1.15, −0.63), and continued to bear the highest disease burden, with mean values exceeding 1900 per 100 000.

At the national level, substantial heterogeneity was found across countries. Regarding DALY rates, Cuba (EAPC = –5.76; 95% UI = −6.04, −5.48) and Belarus (EAPC = –5.14; 95% UI = −5.94, −4.35) recorded the most pronounced declines, whereas Sao Tome and Principe (EAPC = 1.59; 95% UI = 1.05, 2.23) and Sierra Leone (EAPC = 1.60; 95% UI = 1.19, 1.98) showed upward trends. Regarding death rates, Cuba (EAPC = –5.87; 95% UI = −6.15, −5.86) and Serbia (EAPC = –4.69; 95% UI = −4.96, −4.43) experienced the most rapid decreases, while Sierra Leone and Paraguay demonstrated increases (EAPC = 1.69; 95% UI = 1.27, 2.08). For incidence rates, Indonesia and Ethiopia showed the sharpest declines (EAPC = –4.00; 95% UI = −4.29, −3.90), whereas Paraguay and Lesotho exhibited moderate increases (EAPC = 0.90; 95% UI = 0.77, 0.99). For prevalence rates, Indonesia and Iran showed the steepest reductions (EAPC = –5.00; 95% UI = −5.29, −4.83), while Botswana and Lesotho displayed slight increases (EAPC = 0.70; 95% UI = 0.63, 1.02).

Between 1990 and 2023, road injury-related disease burden decreased most rapidly in GNI-UM countries, improved steadily in GNI-LM countries, but declined only marginally in GNI-L countries, with some evidence of apparent rebound in modelled estimates. Regionally, the most substantial declines were observed in Latin America, Eastern Europe, and Western Asia, while several countries in sub-Saharan Africa showed upward trends (**Table 1**; Figures S1–5 and Tables S2–4 in the **Online Supplementary Document**).

**Table 1:** DALYs of road injury cases and rates*

Location	DALYs, n (95% UI)		Proportional change	Rate (95% UI)		EAPC (95% UI)
	**1990**	**2023**		**1990**	**2023**	
All	210.44 (135.52, 296.69)	165.47 (109.63, 232.42)	–0.21	1211.28 (780.02, 1707.71)	770.46 (510.45, 1082.2)	–1.51 (–1.56, –1.46)
GNI-L	31.39 (17.41, 48.84)	59.17 (37.56, 84.44)	0.88	2459.89 (1364.14, 3828.22)	2030.53 (1289.15, 2897.95)	–0.89 (–1.15, –0.63)
GNI-LM	70.86 (45.28, 100.33)	70.86 (45.54, 102.41)	0	905.82 (578.84, 1282.45)	616.59 (396.23, 891.09)	–1.27 (–1.35, –1.18)
GNI-UM	108.19 (72.83, 147.52)	35.44 (26.53, 45.57)	–0.67	1307.56 (880.16, 1782.79)	501.26 (375.19, 644.52)	–2.87 (–3.14, –2.59)

DALY – disability adjusted life year, EAPC – estimated annual percentage change, GNI-L –low-income countries, GNI-LM –lower-middle-income countries, GNI-UM –upper-middle-income countries, UI –uncertainty interval

*Numbers indicate total DALYs (in thousands). Rates are expressed per 100 000 population aged 0–19 y.

### Etiology

In 2023, the cause composition of road traffic injuries showed distinct patterns in DALYs, mortality, incidence, and prevalence, with variations by national income level.

Across all burden indicators (DALYs, deaths, incidence, and prevalence), motor vehicle-related road injuries consistently accounted for the largest proportion worldwide, followed by pedestrian or cyclist injuries, depending on the specific metric. At the global level, motor vehicle-related road injuries accounted for approximately 40.51% of DALYs, 40.63% of deaths, 35.73% of incident cases, and 31.05% of prevalence cases.

Patterns were largely similar across different GNI categories. In contrast, the proportion of motorcyclist road injuries (28.87%) slightly exceeded pedestrian injuries (28.06%) in terms of deaths in GNI-UM countries. In GNI-L countries, motor vehicle-related road injuries were even more dominant, accounting for 47.18% of incidence and 39.94% of prevalence, followed by pedestrian injuries (Figure S6 in the **Online Supplementary Document**).

### Age-specific trends

For DALYs and deaths, PC across age groups showed a consistent downward trend globally, but marked disparities were observed across GNI categories. Specifically, both GNI-L and GNI-LM countries demonstrated increasing trends, whereas GNI-UM countries exhibited declining trends.

For incidence, PC across age groups declined overall. In GNI-L countries, however, incidence increased overall, while in GNI-UM countries it declined. In GNI-LM countries, incidence decreased in the <5 years and 5–9 years groups but increased in the 10–14 years and 15–19 years groups.

For prevalence, PC declined overall in both GNI-LM and GNI-UM countries. In GNI-L countries, prevalence decreased in the <5 years and 5–9 years groups but increased in the 10–14 years and 15–19 years groups. Notably, the 15–19 years group in GNI-L countries showed significant increases across all metrics, with PCs of 1.28 (DALYs), 1.29 (deaths), 0.93 (incidence), and 0.36 (prevalence), respectively. By contrast, the 5–9 years group in GNI-LM countries showed significant decreases in DALYs (PC = –0.30) and deaths (PC = –0.29).

For EAPC, age-specific declining trends were consistent across DALYs, deaths, incidence, and prevalence in all age groups and GNI categories. Of note, the 5–9 years group in GNI-UM countries exhibited significant decreases (DALYs EAPC = –2.20; deaths EAPC = –2.19). Similarly, the <5 years group in GNI-UM countries showed a marked decline in incidence (EAPC = –4.29) (Figure S7 in the **Online Supplementary Document**).

### Sex-specific trends

For age-specific ratios (females *vs.* males), consistent patterns were observed in DALYs and deaths. Males accounted for the majority across all age groups, and the ratio gradually declined with increasing age. For incidence and prevalence, the results were also consistent. Females predominated in the <5 years group, whereas males predominated in the 15–19 years group, with the ratio decreasing progressively with age (Figure S8 in the **Online Supplementary Document**).

### Joinpoint analysis

From 1990 to 2023, the global burden of road traffic injuries declined overall, but with substantial differences across GNI levels. DALYs declined (average annual percentage change (AAPC) = –2.96), with the steepest reductions observed in high-income and GNI-UM regions, particularly during 2015–2020 (annual percentage change (APC) = –6.10). In contrast, GNI-L regions experienced a rebound after 2011 (APC = 1.53 during 2011–2017; APC = 0.78 during 2017–2023). Deaths decreased globally (AAPC = –2.90), with the sharpest reductions in GNI-UM countries during 2015–2020 (APC = –6.02). GNI-L regions showed a decline between 1999–2004 (APC = –3.01) but exhibited an increasing trend in 2017–2023 (APC = 0.86). Incidence fell markedly (AAPC = –2.43), with the fastest reductions in GNI-UM countries during 2006–2015 (APC = –3.73). In GNI-L regions, incidence declined substantially before 2018 (APC = –3.57) but slowed during 2018–2023 (APC = –0.99). Prevalence also decreased significantly (AAPC = –3.06), with the steepest reductions in GNI-UM regions during 2001–2012 (APC = –5.56), and in GNI-L regions during 2010–2018 (APC = –3.62), though the decline moderated in 2018–2023 (APC = –0.93) (Figure S9 in the **Online Supplementary Document**).

From 1990 to 2023, DALY, mortality, incidence, and prevalence rates of road injuries declined in both sexes, but the burden remained higher in males. For DALYs AAPCs = –1.26, for mortality APC = –1.22, for incidence APC = –2.08, and for prevalence APC = –2.57. Females showed more sustained and pronounced declines (*e.g.* DALY APC = –2.29 in 1996–2010), while declines in males slowed markedly after 2018 (*e.g.* DALY APC = –0.29 in 2018–2023). Similar slowdown patterns were revealed for mortality, incidence, and prevalence among males in the most recent period (Figure S10 in the **Online Supplementary Document**).

### Correlation with GNI

**Figure 1:**
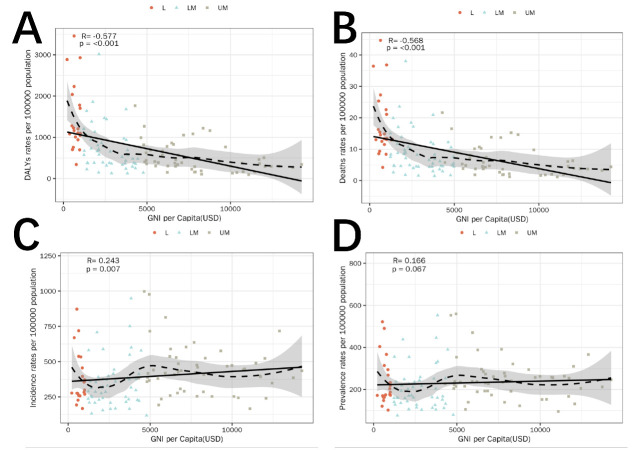
GNI correlation analysis – low income, lower middle income, upper middle income. **Panel A.** Correlation between GNI *per capita* and DALY rates. **Panel B.** Correlation between GNI *per capita* and mortality rates. **Panel C.** Correlation between GNI *per capita* and incidence rates. **Panel D.** Correlation between GNI *per capita* and prevalence rates. Each dot represents one country. The fitted line indicates the linear regression trend. Pearson correlation coefficients (r) and two-sided *P*-values are shown in each panel.

Correlation analysis between GNI *per capita* and the burden of road traffic injuries revealed a significant negative association for both DALYs (Pearson correlation coefficient (r) = –0.577, *P* < 0.001) and mortality (r = –0.568, *P* < 0.001). The incidence exhibited a weak positive correlation with GNI (r = 0.243, *P* = 0.007). This association may reflect differences in surveillance, reporting, and access to the health system, rather than the true underlying risk. Therefore, it should be interpreted cautiously and not as evidence of causality. No statistical significance was revealed in prevalence (r = 0.166, *P* = 0.067), showing an overall flat trend. Notably, some GNI-LM countries exhibited lower incidence and prevalence rates than expected (**Figure 1**).

At the national level, GNI-L countries, such as the Democratic Republic of the Congo, Rwanda, and Angola, showed higher-than-expected DALY and mortality rates, whereas GNI-UM countries, including China, Costa Rica, and the Maldives, exhibited lower-than-expected burdens. For incidence, GNI-UM countries, such as Argentina, Libya, and Egypt, had higher-than-expected rates, while GNI-L countries, including Nepal and Kenya, showed lower-than-expected levels. For prevalence, Egypt, Iran, and Argentina showed notably higher levels than expected, whereas Nepal, Gambia, and the Maldives showed substantially lower levels.

Overall, DALYs and mortality rates declined steadily with increasing economic level, showing a stable negative correlation with GNI, while variations in incidence and prevalence were likely more influenced by national surveillance capacity and reporting quality, with several countries exhibiting lower-than-expected burdens (Figure S11 in the **Online Supplementary Document**).

### Decomposition analysis

**Figure 2:**
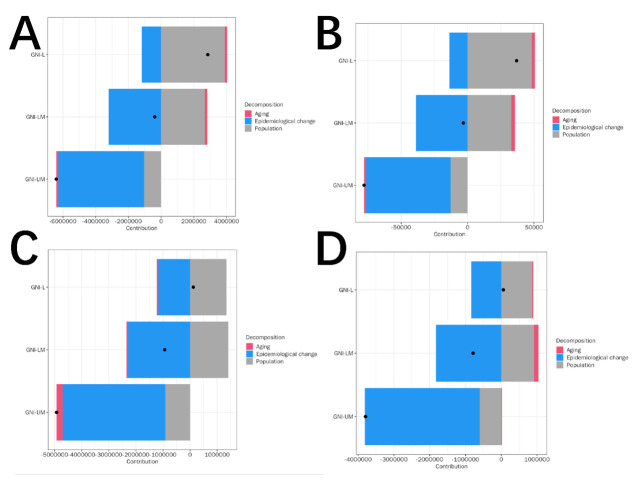
Decomposition analysis-region. **Panel A.** Decomposition of changes in DALYs. **Panel B.** Decomposition of changes in mortality. **Panel C.** Decomposition of changes in incidence. **Panel D.** Decomposition of changes in prevalence. Each bar represents the relative contribution of population growth, age structure, and epidemiological change between 1990 and 2023.

Decomposition analysis revealed that in GNI-L and GNI-LM countries, increases in DALYs and deaths were primarily driven by population growth, with only minor contributions from ageing, while improvements in epidemiology contributed negatively to burden. In contrast, in GNI-UM countries, overall declines in DALYs and deaths were mainly attributable to epidemiological improvements. For incidence and prevalence, increases in GNI-L and GNI-LM countries were largely driven by population growth and partially offset by improvements in epidemiology. In GNI-UM countries, both indicators showed overall declines, with epidemiological improvements contributing most significantly. Sex-stratified results suggested that women were more affected by ageing, whereas reductions among men were more strongly attributable to epidemiological improvements. In summary, increases in burden in GNI-L countries were mainly driven by population growth, while reductions in GNI-UM countries were predominantly due to epidemiological improvements, with notable sex differences (**Figure 2**; Figures S12–14 in the **Online Supplementary Document**). In the study cohort aged 0–19 years, ageing should be interpreted as changes in the age structure within this range (*e.g.* a higher proportion of older adolescents) rather than biological ageing.

### ARIMA model

**Figure 3:**
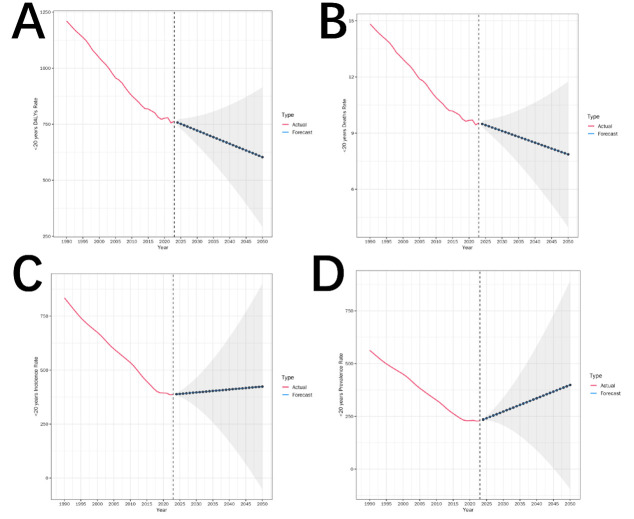
ARIMA forecast. **Panel A.** Forecasted DALY rates. **Panel B.** Forecasted mortality rates. **Panel C.** Forecasted incidence rates. **Panel D.** Forecasted prevalence rates. Solid lines represent observed trends from 1990 to 2023. Dashed lines indicate projected values based on ARIMA models. Shaded areas represent 95% prediction intervals.

Under the assumption that historical trends persist, the ARIMA model suggested a possible rebound in the disease burden by 2050. This projection should be interpreted cautiously, given the inherent uncertainty in GBD estimates. Scenario-based extrapolations from the ARIMA model suggested that, under a trend-continuity assumption, the trauma-related disease burden among individuals under 20 years may continue to decline globally. DALY and mortality rates have shown steady declines since 1990 and are projected to fall <600 per 100 000 and 8 per 100 000, respectively, by 2050. In contrast, incidence has plateaued since 2020 and is projected to rise slightly to 420–450 per 100 000 by 2050, while prevalence reached its lowest point around 2020 and is projected to rebound gradually, exceeding 500 per 100 000 by 2050. The Ljung-Box test confirmed white noise residuals and strong model stability and fit (**Figure 3**; Table S5 in the **Online Supplementary Document**).

## DISCUSSION

In this study, we comprehensively assessed long-term patterns in the burden of road traffic injuries among children and adolescents aged 0–19 years in LMICs, based on estimates from the GBD 2023 study. The results suggested overall declines in estimated DALYs, mortality, incidence, and prevalence between 1990 and 2023. Differences in these estimates across income levels, age groups, and sexes were consistently observed. Importantly, all findings should be interpreted as exploratory patterns in modelled estimates rather than directly observed epidemiological changes, particularly in LMICs where primary data are limited.

### Overall trends and interpretation of modelled estimates

The observed declines in DALYs and mortality are broadly consistent with prior GBD analyses and may reflect improvements in trauma care, road-safety legislation, and injury-prevention measures across many regions. However, Joinpoint analyses suggested that these declines were not uniform across time or income groups. In particular, the apparent slowing or reversal of declining trends in some low-income countries should be interpreted cautiously. Such patterns may partly reflect changes in data inputs, covariate availability, or model recalibration over time, rather than true increases in injury risk.

By contrast, upper-middle-income countries exhibited more pronounced and sustained declines in most metrics, which aligns with previous global injury studies using GBD data [20–23]. These differences likely reflect structural heterogeneity captured indirectly by the models, such as variation in infrastructure investment, enforcement of traffic regulations, and access to emergency and rehabilitative care. Nonetheless, because these factors are not directly measured in the present analyses, causality cannot be inferred.

### Etiological patterns and injury mechanisms

Across all income groups, motor vehicle-related injuries accounted for the largest proportion of estimated DALYs, mortality, incidence, and prevalence. This finding is consistent with the GBD classification of road injuries and reflects the dominant contribution of motorised transport to the disease burden. Differences in the relative contributions of motor vehicle, motorcyclist, pedestrian, and cyclist injuries across different income levels may reflect varying transport structures and exposure patterns, as represented in the underlying data sources and covariates used by GBD. However, given the modelled estimates used, these findings should be viewed as indicative rather than definitive to explain the mechanism of injury in specific settings [24].

### Age- and sex-specific analysis

Age- and sex-stratified analyses consistently showed a higher estimated burden among males and older adolescents, particularly those aged 15–19 years. This pattern is consistent with prior injury epidemiology literature and likely reflects differences in exposure and injury mechanisms [25]. For instance, a higher proportion of older adolescents, especially males, serve as drivers or motorcyclists. Differences were observed between PC and EAPC in some age groups and regions. PC primarily reflects changes in the absolute number of cases, whereas EAPC indicates temporal trends in age-standardised rates. Accordingly, these measures reflect different dimensions of disease burden and do not necessarily imply conflicting trend patterns. EAPC and joinpoint regression were used to project changes in the burden of road traffic injuries over time.

### Income level, ecological associations, and heterogeneity

The negative ecological associations observed between GNI *per capita* and estimated DALY and mortality rates are consistent with previous global health research. Nonetheless, they should be interpreted strictly as descriptive associations rather than evidence of causality. National income reflects a wide range of contextual factors, including infrastructure quality, health system capacity, enforcement intensity, and political stability, which are not explicitly modelled in this study. Similarly, the weak positive association between GNI and incidence may reflect differences in detection, reporting, and data availability rather than true variations in injury risk.

While stratified analysis by GNI could compare the disease burden across income levels, it inevitably masks substantial within-group heterogeneity, particularly among low-income countries affected by conflict, humanitarian crises, or rapid urbanisation [26]. Consequently, these findings suggested broad inequities rather than providing guidance for policy decisions in specific countries.

### Decomposition analysis within the GBD framework

Decomposition analysis within the GBD modelling framework was used as an explanatory tool to illustrate how population growth, changes in age structure, and epidemiological changes contributed to the estimated disease burden. In low-income countries, population growth emerged as the dominant contributor to increasing the estimated burden, whereas epidemiological improvements played a crucial role in upper-middle-income countries [27]. In this study population, ageing should be interpreted as changes in the age structure within the 0–19 years range, such as a higher proportion of older adolescents, rather than biological ageing.

Because decomposition relies on model-derived inputs and assumptions, the relative contributions of each component should be interpreted cautiously and viewed as illustrative rather than definitive [28]. Interactions between demographic and epidemiological factors are possible, and these cannot be fully resolved within the current study.

### Scenario-based extrapolations using ARIMA models

Exploratory ARIMA models were used to project future patterns under the assumption of trend continuity. These projections suggested continued declines in DALYs and mortality, while the incidence and prevalence would stabilise or modestly increase. Importantly, these findings should not be interpreted as exploratory projections of future burdens. Projections based on modelled estimates from GBD using time-series methods increase uncertainty and do not account for potential policy changes, technological advances, conflict, climate change, or shifts in transport systems in the future [25].

The difference between projected mortality and incidence patterns reflects a well-recognised phenomenon in injury epidemiology – improvements in survival and trauma care may reduce fatal outcomes while increasing the number of individuals living with injury-related disability. However, our findings remain speculative and should be treated as exploratory, scenario-based projections rather than empirical forecasts.

### Comparison with previous research

Previous GBD studies have consistently reported a substantial burden of road traffic injuries across all age groups, with particularly high mortality and disability in LMICs. Earlier studies have shown that the mortality of road injuries has increased or plateaued during the 1990s and early 2000s, reflecting rapid motorisation and limited safety facilities in many low-resource regions [22,29]. More recently, the mortality and DALYs have stabilised or gradually declined, possibly due to expanded road safety initiatives and improvements in trauma care [26].

Our findings, based on modelled estimates from GBD 2023, are broadly consistent with these more recent reports, showing that DALY and mortality rates among children and adolescents have declined since 1990. However, this study focused specifically on children and adolescents (aged 0–19 years) within LMICs and examined heterogeneity across income levels, age groups, and sexes. Our findings revealed that the estimated disease burden in low-income countries was the highest, and declines in the disease burden appeared less pronounced and less stable than those in upper-middle-income regions.

Compared with earlier studies based on GBD that primarily reported global or regional aggregates, the present study performed decomposition analysis to illustrate the contributions of population growth, changes in age structure, and epidemiological change to the estimated burden. Our results suggest that in low-income countries, population growth may offset epidemiological improvements captured by the models. In contrast, in upper-middle-income countries, estimated reductions in the disease burden are more attributable to epidemiological change. These findings are directionally consistent with prior global health research, highlighting the roles of population growth and health system capacity in shaping the burden of road injuries. Nonetheless, our findings should be interpreted cautiously, given that they were derived from modelled estimates.

Some earlier studies based on GBD have indicated that the incidence of road traffic injuries increases while the mortality decreases. On the contrary, our analysis suggested relatively stable or modest increases in the incidence and prevalence of road traffic injuries in recent years, particularly in lower-income countries. This inconsistency in findings likely reflects differences in data availability, surveillance sensitivity, and modelling assumptions rather than true epidemiological changes. As with previous GBD-based injury research, the estimated incidence and prevalence of road traffic injuries are especially sensitive to changes in case detection and survival rates. Hence, the results of trends across studies and time periods should be interpreted with caution.

Overall, while the present findings align with the general trajectory reported in prior GBD analyses, they underscore persistent inequalities in the estimated burden of road traffic injuries among children and adolescents in LMICs [30–32]. Rather than contradicting previous conclusions, this study provides complementary evidence of the contributions of population growth and income levels to the burden of road traffic injuries in younger populations.

### Limitations

First, all analyses were based on modelled estimates from the GBD 2023 study, rather than directly observed data. Although GBD provides the most comprehensive and standardised estimates on global road traffic injuries, its outputs are inherently influenced by data availability, covariate selection, and modelling assumptions. This limitation is particularly evident in low-income countries, where primary injury surveillance systems are often incomplete or absent, and estimates are more likely to be modelled based on covariates. Consequently, changes in the disease burden should be interpreted as modelled estimates rather than definitive epidemiological changes.

Second, stratified analysis by age, sex, country, and income level increases the risk of false-positive findings due to multiple comparisons. To mitigate this risk, FDR correction was performed where appropriate. Furthermore, we prioritised long-term trends (EAPC and joinpoint regression) and consistency across methods and uncertainty intervals over isolated statistically significant results. Nevertheless, some findings for specific subgroups should be interpreted cautiously.

Third, ecological analysis based on GNI *per capita* is subject to ecological fallacy. GNI was used as a broad contextual indicator rather than a causal determinant, and it cannot capture critical factors such as road infrastructure quality, enforcement intensity, health system capacity, urbanisation, or conflict. Accordingly, associations between GNI and the burden of road traffic injuries should be viewed as descriptive and hypothesis-generating rather than definitive.

Fourth, the decomposition analysis was conducted entirely within the GBD modelling framework and relied on model-derived inputs. The partitioning of burden changes into population growth, age structure, and epidemiological change is therefore illustrative rather than definitive. The relative contributions may be sensitive to underlying modelling choices. In this study, ageing in the 0–19 years age range reflects shifts in the age structure within this range (*e.g.* a higher proportion of older adolescents) rather than biological ageing, and interactions between components cannot be fully disentangled.

Fifth, ARIMA models were used for exploratory, scenario-based projections of future trends, assuming trend continuity. Projections based on modelled estimates from GBD using time-series methods increase uncertainty and do not account for potential policy changes, technological advances, urbanisation, conflict, or climate-related factors in the future. These projections should therefore not be interpreted as actual future burden, but rather as illustrative projections based on historical patterns.

Finally, countries were divided into broad GNI categories, which may mask substantial within-group heterogeneity, particularly among low-income countries affected by conflict, humanitarian crises, or rapid socioeconomic transition. Therefore, the findings of this study serve more as a reference for global and comparative analyses than as a guide for specific policy decisions within individual countries. Future studies incorporating improved primary surveillance data and country-level analyses are needed to better distinguish true epidemiological change from modelling estimates.

### Policy priorities and the imperative to strengthen LMIC capacity

Addressing the burden of road injuries among children and adolescents in LMICs is both an urgent health priority and a matter of social equity. These regions face the greatest burden due to inadequate infrastructure, weak trauma care systems, and fragile health services. Although global estimates show overall declines in mortality and DALYs, our findings indicate only modest progress in GNI-L countries, with signs of stagnation and apparent rebound in modelled estimates, underscoring their disproportionate vulnerability. Conflict-affected regions further illustrate how structural deficits, such as disrupted supply chains and insufficient emergency capacity, exacerbate health inequities, particularly for children [33].

To address this gap, political commitment and targeted policies are essential. Strengthening surveillance and screening systems can help identify high-risk populations and generate real-time, high-quality data, thus reducing reliance on modelled estimates. Governments and international organisations should prioritise equitable resource allocation in trauma care, pediatric services, and rehabilitation. It is also crucial to enforce road safety legislation, including seatbelt and helmet use, speed limits, and pedestrian and cyclist protection, with strategies adapted from high-income countries to local contexts [34]. Beyond the health sector, coordinated cross-sectoral action is needed in transportation, education, urban planning, and law enforcement to tackle upstream determinants. International collaboration – through funding, technical support, and policy transfer – can further enhance LMIC capacity. Ultimately, sustainable progress relies on strong political will, equity-focused governance, and the protection of vulnerable groups, especially children and adolescents [35].

## CONCLUSIONS

This analysis of modelled estimates from GBD 2023 suggests that the burden of road traffic injuries among children and adolescents in LMICs has declined over the past three decades, while there are substantial differences by income level, age group, and sex. Hence, it is necessary to continue to invest in road safety, trauma care, and injury prevention, particularly in low-income regions. Future research should incorporate improved surveillance data on road injuries and country-level analyses to better distinguish true epidemiological changes from modelling estimates and to inform the development of more targeted policy interventions.

## Additional material


Online Supplementary Document
