# Supplementary material for: Trends in the burden of road traffic injuries among children and adolescents aged 0–19 years in low- and middle-income countries, 1990–2023
Source: J Glob Health. 2026 Mar 13;16:04094. doi: 10.7189/jogh.16.04094 (PMC12981736; doi:10.7189/jogh.16.04094)
Supplement: Online Supplementary Document [file jogh-16-04094-s001.pdf]

Supplement to: Song Z, Zhang B, Pang S, Qiu M, Huang J, Hao J, Yang X, Li Y. Trends in the burden of road traffic injuries among children and adolescents aged 0–19 years in low- and middle-income countries, 1990–2023. J Glob Health. 2026;16:04094.

Table S1. Adherence to JoGH's GRABDROP guidelines items.

**JoGH guideline items**
1. Please list all papers published by each co-author in previous 3 years that were based on secondary analysis of a big data repository
**Zhe Song:** none published papers;<br>**Bing Zhang:** 中华危重病急救医学杂志，2025,37(11):1033-1039.DOl:10.3760/cma.j.cn121430-20250306-00219.<br>**Sihan Pang:** none published papers;<br>**Mingliang Qiu:** none published papers;<br>**Jiang Huang:** Scientific Reports. 2024 Oct 18;14(1):24522. doi: 10.1038/s41598-024-75887-z.<br>**Junke Hao:** none published papers;<br>**Xiao Yang:** ①Travel Behaviour and Society. Volume 41, October 2025, 101051. https://doi.org/10.1016/j.tbs.2025.101051<br>②Landscape and Urban Planning. Volume 259, July 2025, 105343. https://doi.org/10.1016/j.landurbplan.2025.105343<br>③2025 28th International Conference on Computer Supported Cooperative Work in Design (CSCWD). DOI:10.1109/CSCWD64889.2025.11033440<br>**Yong Li:** 中华危重病急救医学杂志，2025,37(11):1033-1039.DOl:10.3760/cma.j.cn121430-20250306-00219.
2. Please explain the key elements of your study design and the use of the available datasets that make your study an original scientific contribution
**1. Core innovations in study design:**<br>This study focuses on the burden of road injuries among children and adolescents (0–19 years) in low- and middle-income countries (LMICs). It integrates multi-dimensional stratified analyses (global, regional, and national levels) with joinpoint regression and decomposition methods to identify temporal change points and to quantify the relative contributions of population growth, age structure, and epidemiological change to the observed burden trends.<br>**2. Value of GBD database application:**<br>By fully leveraging the GBD database's strengths of global coverage and long-term time-series estimates, this study provides a comprehensive assessment of incidence, prevalence, mortality, and DALYs related to road injuries from 1990 to 2023. Unlike previous studies that reported only global patterns, this work offers a dedicated LMIC-focused evaluation and uses an integrated analytical framework to explore both trends and underlying drivers of change, thereby contributing original, policy-relevant evidence for global injury prevention and health planning.
3. Please list all publications that addressed similar research questions in the same dataset and indicate where you cited them in your paper
1. **doi: 10.1016/j.pmedr.2025.103051**<br>In the Introduction section, it is cited as:<br>"The burden is disproportionately concentrated in low-income countries, due to inadequate infrastructure, limited healthcare capacity, and weaker road safety regulations[1, 4]."<br>2. **doi: 10.1038/s41591-022-01990-1**<br>In the *Methods section*, it is cited as:<br>"The GBD database is an international collaborative project that provides standardized, comparable estimates of health loss from major diseases and injuries across countries and time[8]."<br>3. **doi: 10.1016/j.aap.2017.09.013**<br>In the Introduction section, it is cited as:

"Road traffic injuries are a major global public health concern, imposing substantial health, social, and economic burdens[1, 2]."

4. **doi: 10.3389/fneur.2025.1526524**
   In the Statistical analysis section, it is cited as:
   "Future trends in mortality and DALYs were projected using an ARIMA model, which integrates autoregression (AR), differencing (I), and moving average (MA)…[18]."
5. **doi: 10.1136/ip-2024-045536**
   In the Discussion section, it is cited as:
   "However, Joinpoint analysis indicates that these improvements are neither consistent across regions nor sustained over time[20]."
6. **doi: 10.1016/j.jot.2024.03.002**
   In the Discussion section, it is cited as:
   "In contrast, in GNI-UM countries, the rise in motorcycle injuries reflects the increasing affordability of two-wheeled vehicles and insufficient helmet use[23]."
7. **doi: 10.1016/s2215-0366(21)00395-3**
   In the Discussion section, it is cited as:
   "The GBD 2019 study found no significant reduction in the burden of mental disorders from 1990 to 2019[24]."
8. **doi: 10.1136/bmj-2024-080969**
   In the Limitations section, it is cited as:
   "For example, discrepancies between modeled estimates and surveillance data have been documented for neglected tropical diseases in China[25]."
9. **doi: 10.1016/s0140-6736(20)30677-2**
   In the Discussion section, it is cited as:
   "These approaches provide a more nuanced understanding of structural and epidemiological drivers…[30]."

4. Please explain how you addressed multiple testing through an appropriately rigorous statistical threshold and indicate this in the methods section

Thank you for your comment regarding multiple testing. In the revised Methods section, we clarified our approach to controlling type I error arising from extensive subgroup and trend analyses.

This study used the Global Burden of Disease (GBD) database and included multiple dimensions of stratification, including age group, sex, income level, region, and outcome (DALYs, mortality, incidence, and prevalence). To reduce the risk of false-positive findings due to multiplicity, we controlled the false discovery rate (FDR) using the Benjamini–Hochberg procedure, with a corrected significance threshold of $q < 0.05$.

For analyses where FDR adjustment is not appropriate (e.g., Joinpoint regression), we relied on the permutation-based significance testing embedded within the Joinpoint framework, which accounts for multiple comparisons across segments.

These details have been explicitly added to the Statistical Analysis subsection of the Methods to ensure transparency and methodological rigor.

5. Please declare to what extent have AI chatbots been used in developing your paper and to which parts of the paper did they contribute

After the main scientific content of the manuscript had been completed, AI-assisted chatbots were used solely to support language polishing and improve clarity of expression in parts of the Introduction and Discussion sections.

No AI tools were used for data extraction, statistical analysis, interpretation of results, or generation of scientific conclusions. All methodological and analytical components were performed and verified by the authors, who take full responsibility for the manuscript.

Table S2. Deaths of road injury cases and rates

location	Number			Rate		
	1990	2023	Percentage change(100%)	1990	2023	EAPC(95% UI)
All location	2.58(1.64~3.66)	2.07(1.36~2.92)	-0.2	14.84(9.42~21.07)	9.65(6.35~13.58)	-1.45 (-1.5 ~ -1.4)
GNI-L	0.39(0.21~0.6)	0.75(0.48~1.06)	0.92	30.35(16.69~47.41)	25.65(16.32~36.55)	-0.83 (-1.1 ~ -0.56)
GNI-LM	0.87(0.55~1.23)	0.88(0.56~1.28)	0.01	11.07(6.99~15.75)	7.68(4.89~11.13)	-1.22 (-1.3 ~ -1.14)
GNI-UM	1.33(0.88~1.82)	0.44(0.33~0.57)	-0.67	16.02(10.59~22.04)	6.26(4.63~8.11)	-2.8 (-3.08 ~ -2.52)

Table S3 Incidence of road injury cases and rates

location	Number			Rate		
	1990	2023	Percentage change(100%)	1990	2023	EAPC(95% UI)
All location	145.1(122.92~168.7)	83.11(70~97.48)	-0.43	835.19(707.53~971.03)	386.99(325.92~453.9)	-2.47 (-2.56 ~ -2.39)
GNI-L	13.46(11.83~15.18)	14.55(12.93~16.22)	0.08	1054.57(927.03~1189.82)	499.4(443.8~556.69)	-2.38 (-2.47 ~ -2.29)
GNI-LM	40.52(34.85~46.87)	33.94(28.78~39.62)	-0.16	517.9(445.43~599.06)	295.35(250.42~344.74)	-1.79 (-1.87 ~ -1.71)
GNI-UM	91.13(76.25~106.66)	34.62(28.29~41.64)	-0.62	1101.34(921.49~1288.98)	489.63(400.06~588.97)	-2.61 (-2.73 ~ -2.49)

Table S4. Prevalence of Road Injury Cases and Rates

location	Number			Rate		
	1990	2023	Percentage change(100%)	1990	2023	EAPC(95% UI)
All location	97.93(88.46~108.53)	49.12(44.88~53.97)	-0.5	563.69(509.14~624.68)	228.74(208.97~251.28)	-3.03 (-3.15 ~ -2.9)
GNI-L	8.64(7.79~9.57)	9.15(8.36~10.03)	0.06	677.01(610.69~750.41)	314(286.9~344.27)	-2.45 (-2.54 ~ -2.37)
GNI-LM	26.42(23.94~29.19)	20.7(19~22.6)	-0.22	337.69(305.97~373.08)	180.15(165.32~196.67)	-2.08 (-2.18 ~ -1.99)
GNI-UM	62.88(56.73~69.77)	19.27(17.52~21.33)	-0.69	759.89(685.58~843.17)	272.57(247.81~301.74)	-3.49 (-3.68 ~ -3.31)

Table S5. ARIMA Forecast

Measures	Parameters	AIC	BIC	Ljung–Box test *p*-value
DALYs ALL	ARIMA(0,2,1)	220.48	223.41	0.458
Deaths ALL	ARIMA(0,2,1)	-62.47	-59.54	0.5015
Incidence ALL	ARIMA(0,2,0)	161.17	162.64	0.6567
Prevalence ALL	ARIMA(0,2,1)	130.34	133.28	0.659

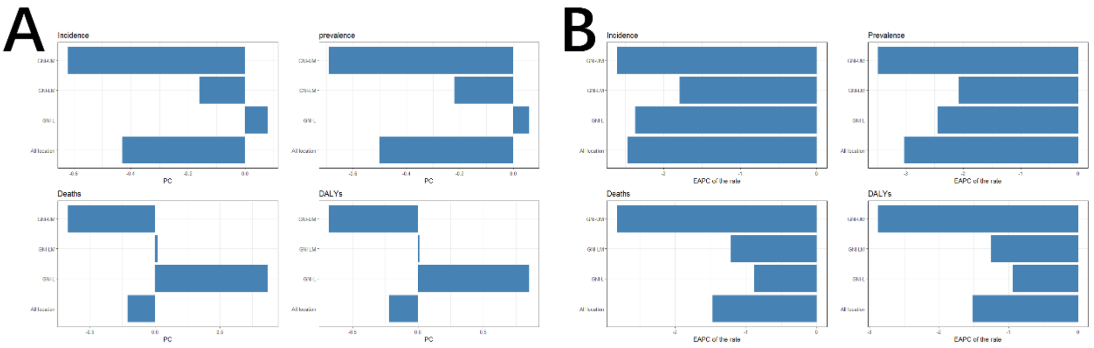

Figure S1. Trends in PC and EAPC

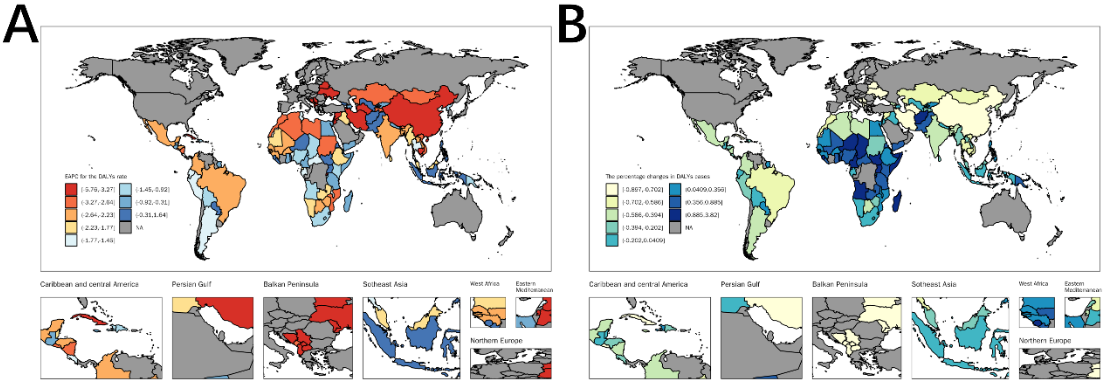

Figure S2. World Map of DALYs

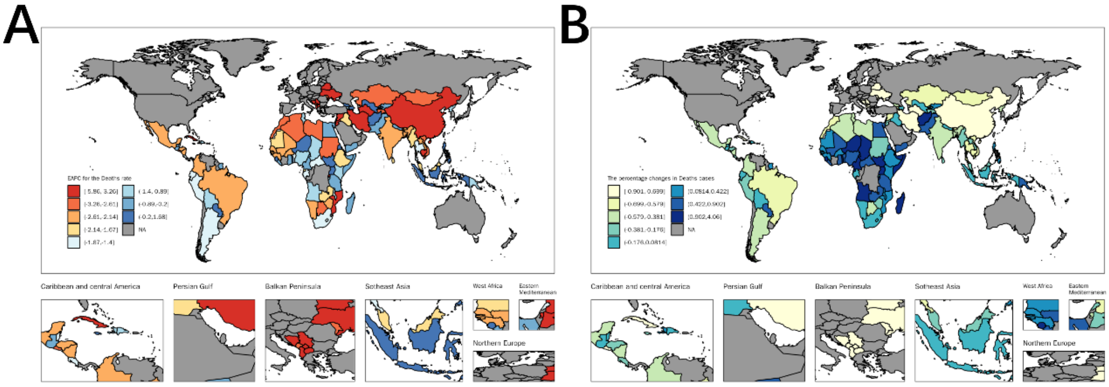

Figure S3. World Map of Deaths

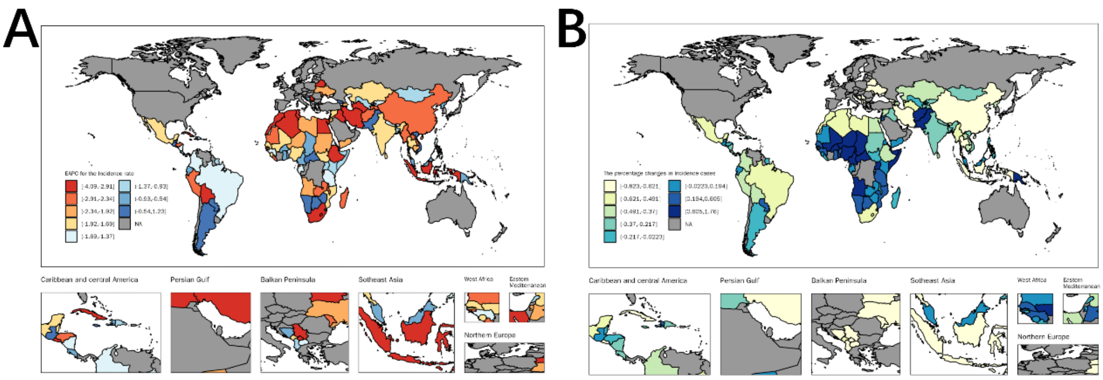

Figure S4. World Map of Incidence

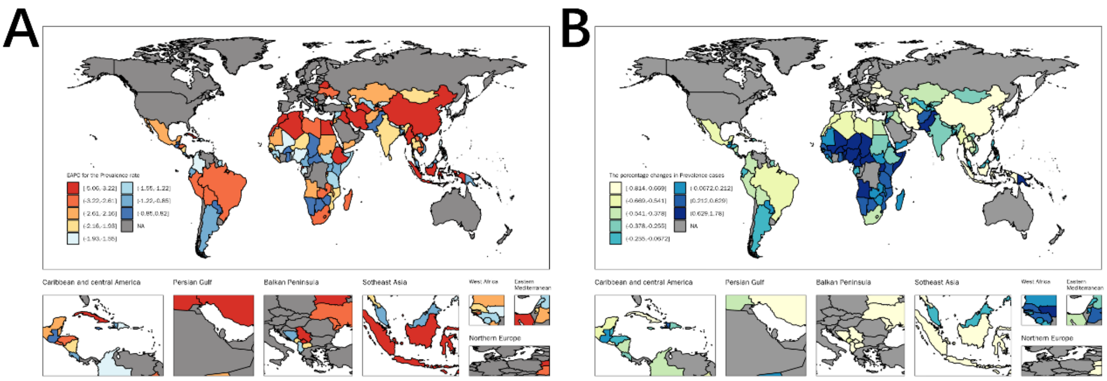

Figure S5. World Map of Prevalence

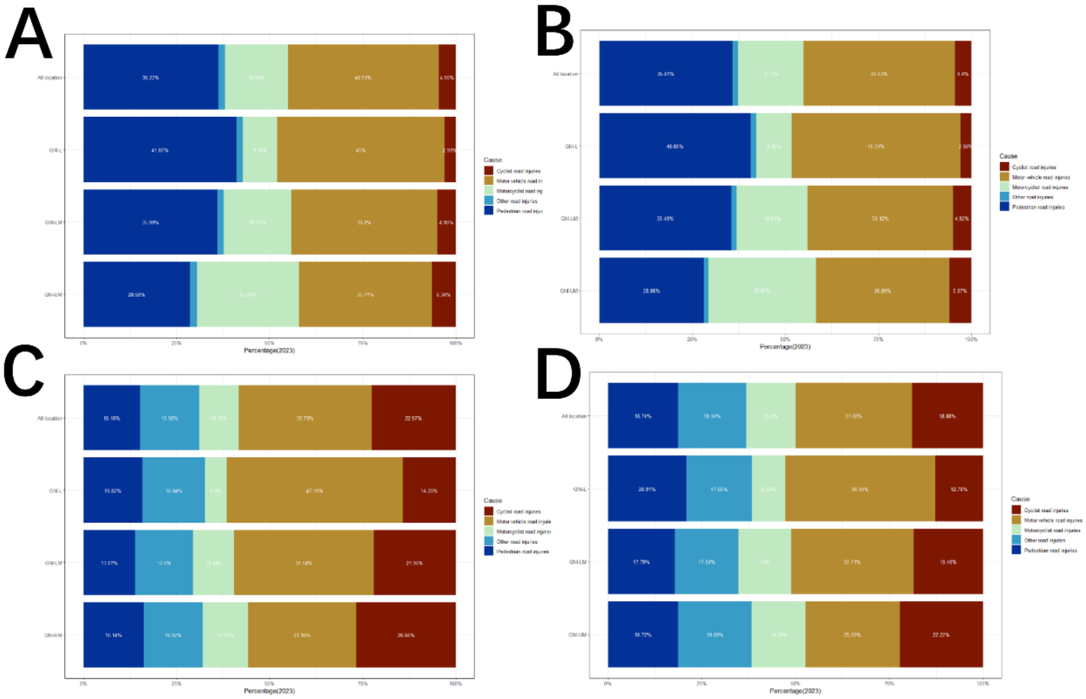

Figure S6. Proportion of Different Etiologies

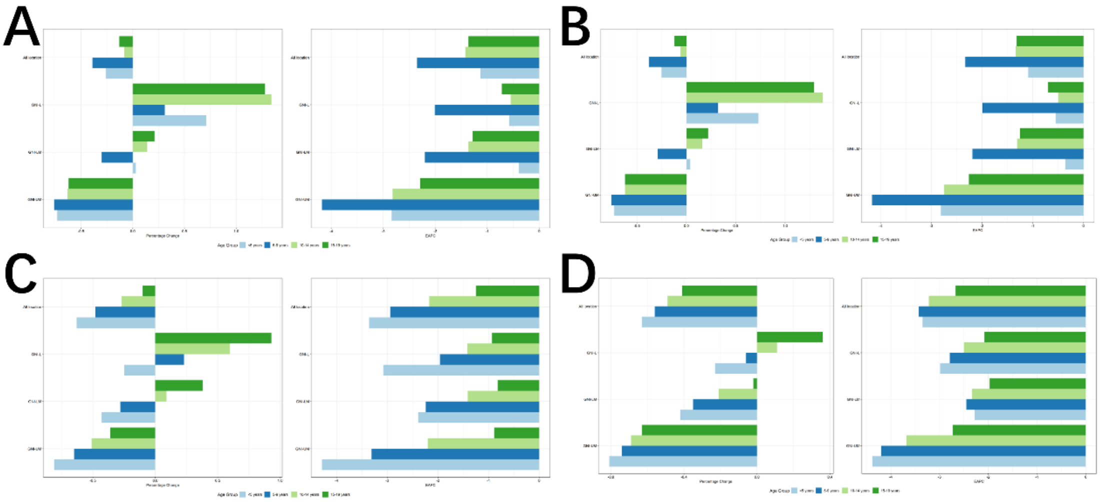

Figure S7. Trends in Different Age Groups

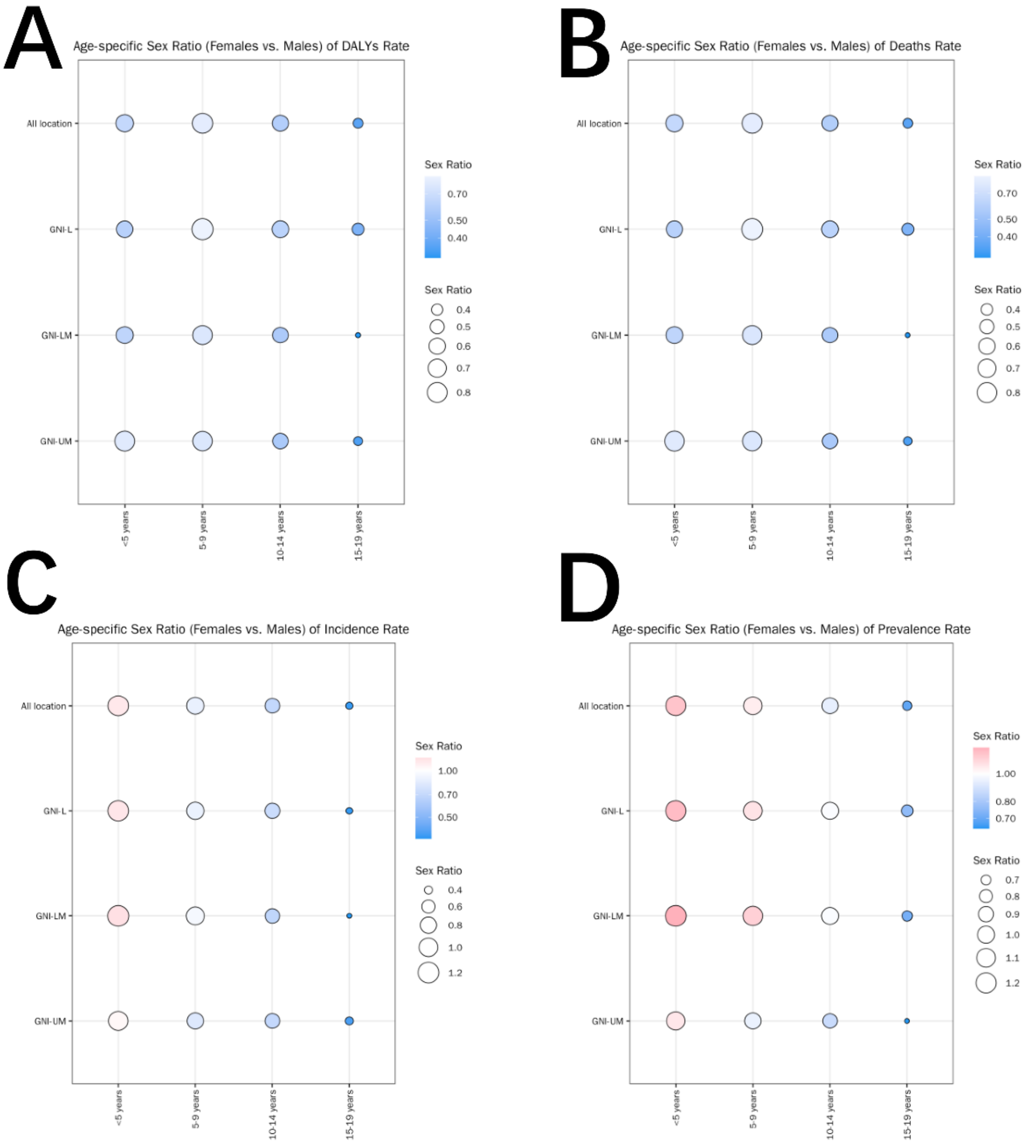

Figure S8. Trends in Sex-Specific Changes

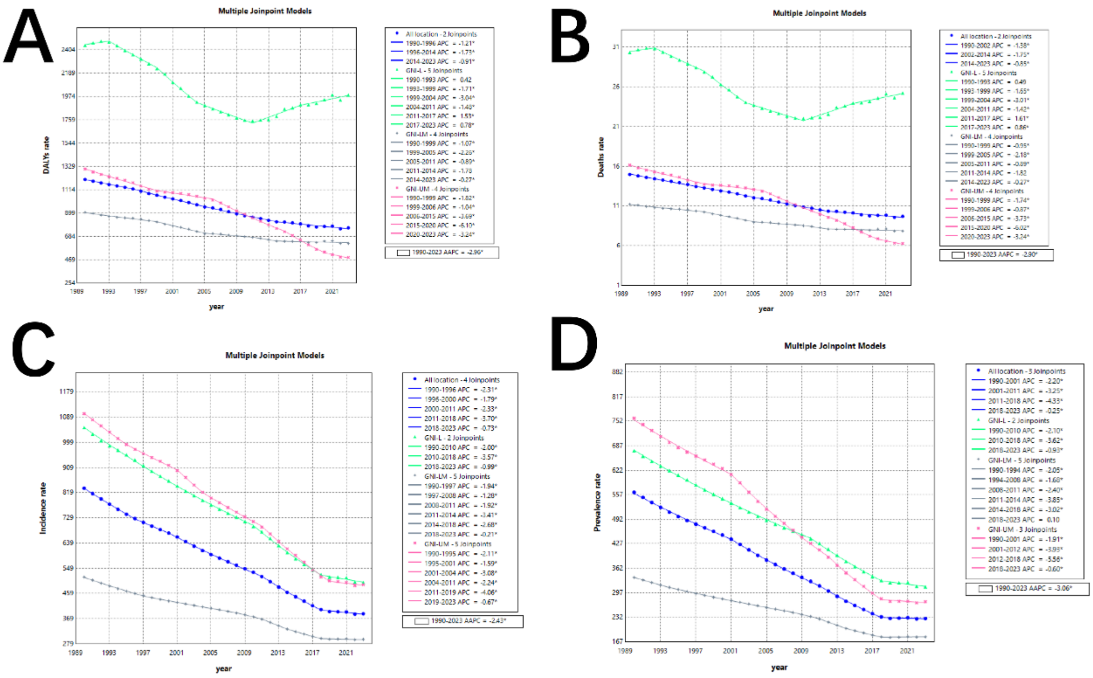

Figure S9. Joinpoint Analysis - Different Regions

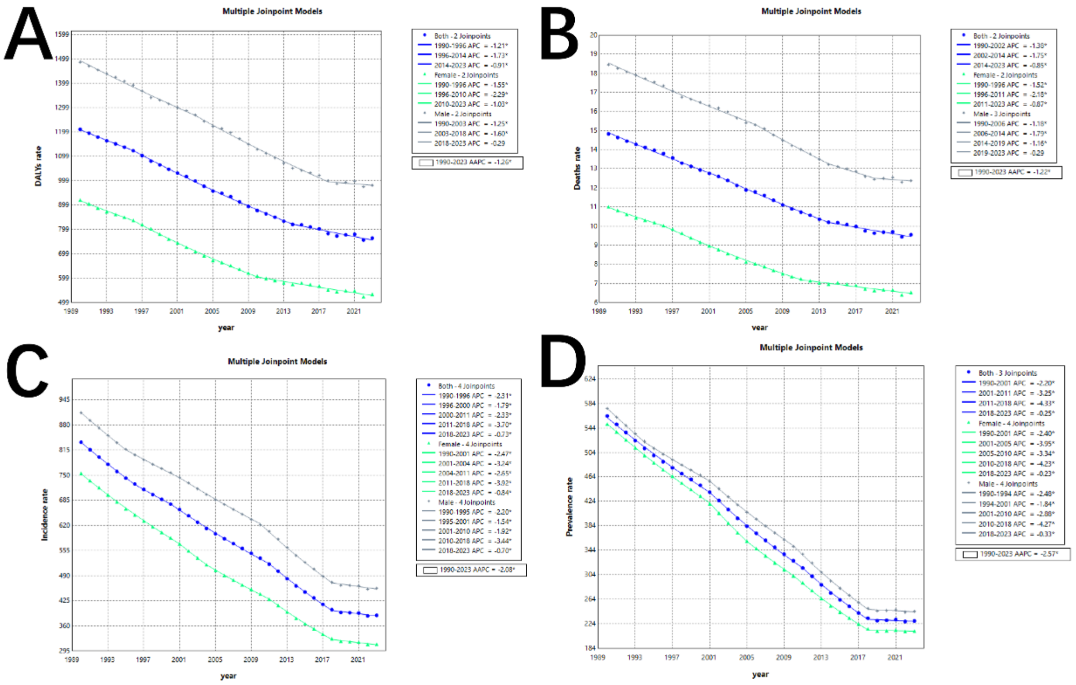

Figure S10. Joinpoint Analysis - Different Sexes

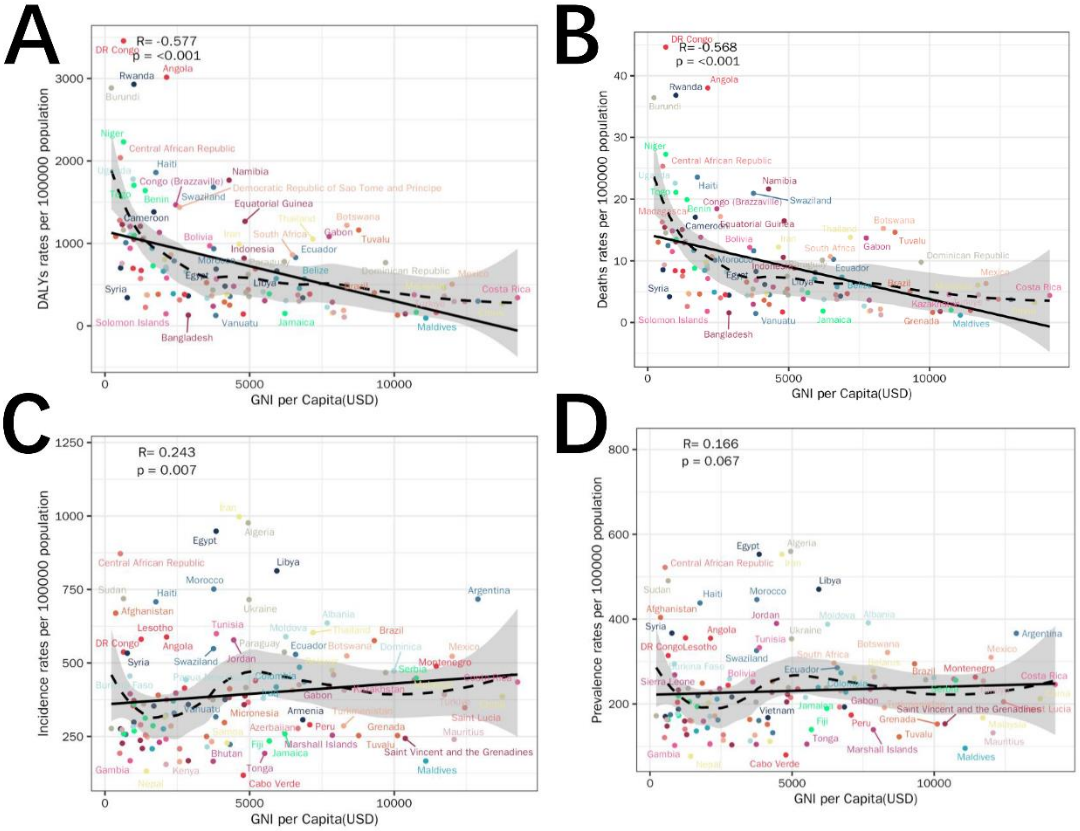

Figure S11. GNI Correlation Analysis - Country

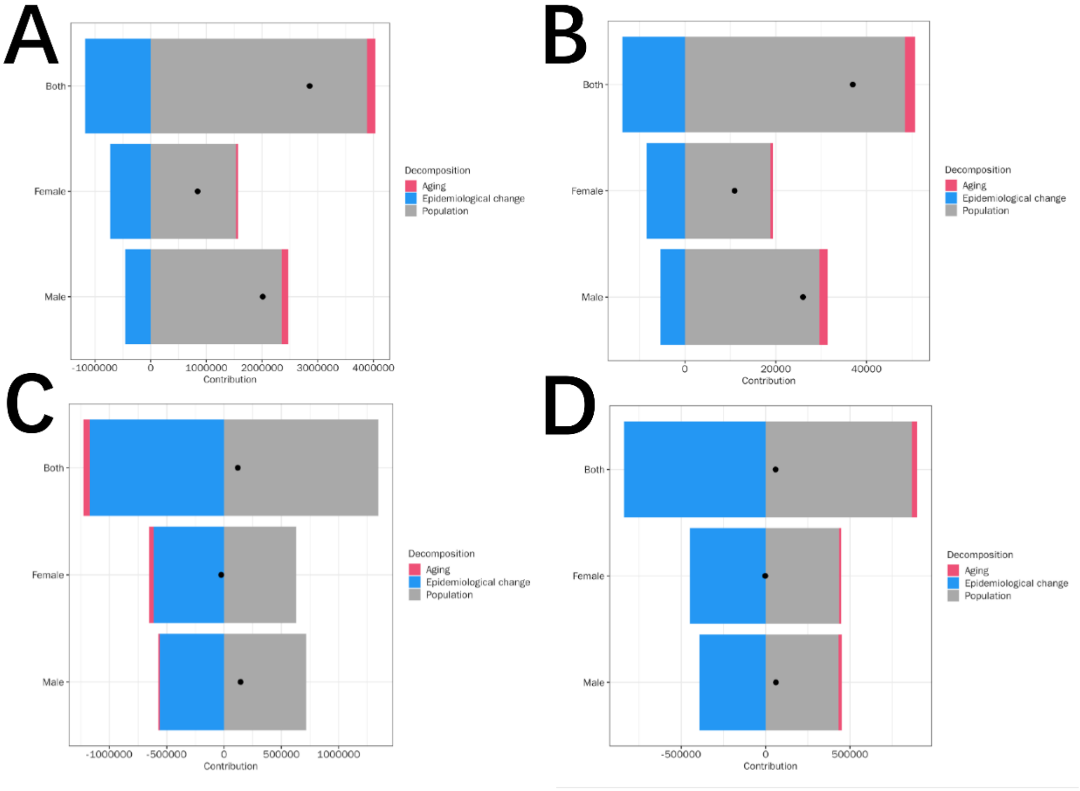

Figure S12. Decomposition Analysis -Sex - GNI-L

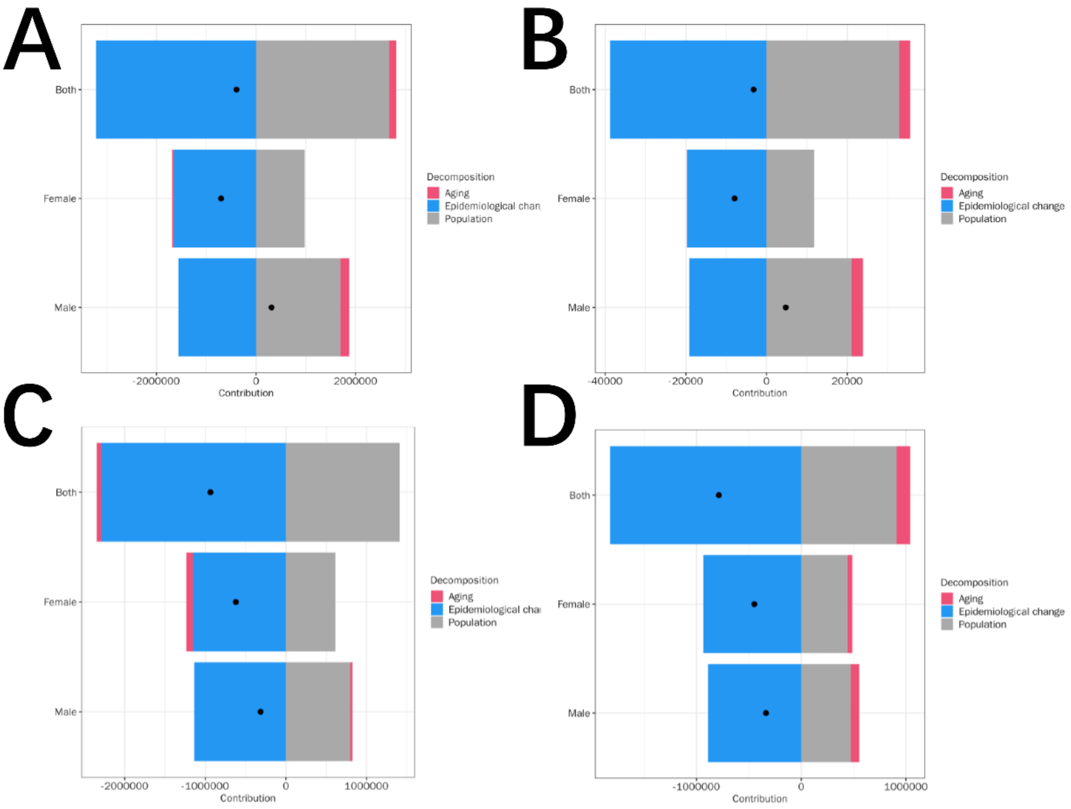

Figure S13. Decomposition Analysis - Sex - GNI-LM

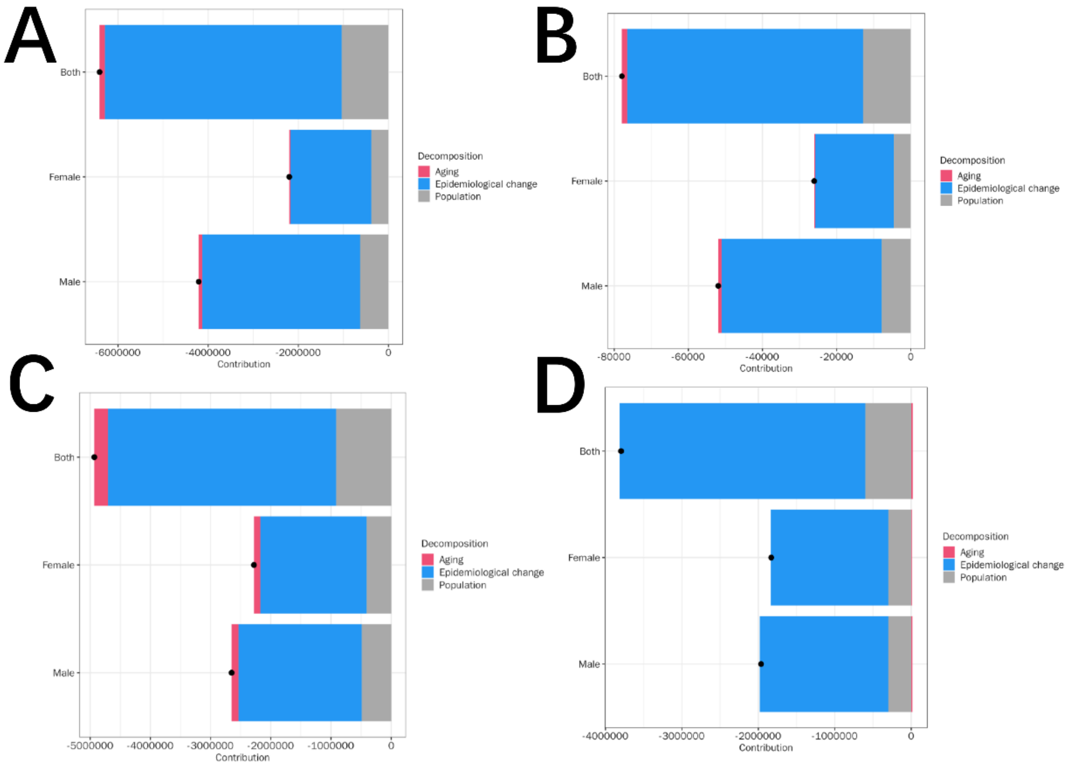

Figure S14. Decomposition Analysis - Sex - GNI-UM
